# New insights into roles of IL-7R gene as a diagnostic biomarker for post-stroke depression

**DOI:** 10.3389/fimmu.2024.1506214

**Published:** 2024-12-23

**Authors:** Mengyu Liu, Haochen Sun, Qun Yao, Duohao Wang, Jihong Zhang, Xing Ye, Xinyang Qi

**Affiliations:** 1 Department of Neurology, Affiliated Nanjing Brain Hospital, Nanjing Medical University, Nanjing, China; 2 Department of Neurology, School of Medicine, Southeast University, Nanjing, China

**Keywords:** stroke, post-stroke depression, major depression, neuroinflammation, IL-7R

## Abstract

**Background:**

Post-stroke depression (PSD) is the most prevalent neuropsychiatric complication following a stroke. The inflammatory theory suggests that PSD may be associated with an overactive inflammatory response. However, research findings regarding inflammation-related indicators in PSD remain inconsistent and elusive. This study aimed to screen the diagnostic markers that helps to distinguish between PSD and post-stroke non-depressed (PSND) patients.

**Methods:**

Two GEO datasets, including patients with major depression disease (MDD) and controls (CON, GSE98793), ischemic stroke (IS) and CON (GSE16561), were used to analyzed differentially expressed genes (DEGs) and perform enrichment analysis. Protein-protein interaction (PPI) network and Random Forest analysis were used to screen the candidate hub genes. CIBERSORT was performed to analyze the immune infiltration. We analyzed the proteins that interact with the hub genes using string database, circRNA-miRNA-mRNA ceRNA network of the hub genes using RNAInter, miRWalk, miRDB and Starbase databases, and the drugs that regulate the hub genes by DSigDB database. We further verified the expression of the hub genes using Quantitative Real-Time PCR from the blood of patients and CON.

**Results:**

From the screened 394 DEGs, the DEGs were found primarily related to activation of immune response. PPI network and random forest analysis obtained the hub genes: IL-7R. ROC analysis showed that IL-7R had a good diagnostic and predictive effect on MDD and IS patients. The proportions of macrophages M0 and monocytes in patients were significantly higher than those in CON. We constructed PPI network and ceRNA network that related to IL-7R. The perturbagen signatures and computational drug signatures were found that can target IL-7R. The expression of IL-7R in MDD, PSND and PSD patients was lower than that in CON, and the expression of IL-7R in PSD patients was lower than that in PSND patients.

**Conclusion:**

These findings indicate that IL-7R may serve as a diagnostic marker to distinguish between PSD and PSND patients, and targeting IL-7R as a therapeutic target could potentially improve treatment outcomes for PSD.

## 1 Introduction

Post-stroke depression (PSD) is a mental disorder characterized by emotional disturbances after a stroke, affecting over 30% of stroke survivors (1). Clinically, patients with PSD can exhibit symptoms such as depressed mood, anhedonia, and slow thinking (2). It compromises patients’ functional recovery, increases the economic and emotional burden for the patients’ family (3). However, PSD involves both neurological and psychiatric factors, which makes its etiology and pathological mechanisms quite complex (4). Therefore, it is critical to find diagnostic biomarkers and improve treatment for PSD.

Neuroinflammation is considered a major cause of secondary damage after stroke, which is believed to play a critical role in the occurrence and development of PSD (5). Once a stroke occurs, astrocytes and microglia as the first line of immune defense are activated, induce cytokines, including interleukin (IL), tumor necrosis factor (TNF), and interferon (4). Then the T cells and the peripheral blood immune cells are recruited and migrate into the brain (6). These cells can also secrete a mass of cytokines, such as ILs (7). The increased levels of cytokines contribute to a decrease in the amount of 5-hydroxytryptamine (5-HT), leading to depression (1). Among the cytokines, ILs are considered the important diagnostic or prognostic biomarkers of PSD (8). Previous studies found that the increased levels of pro-inflammatory cytokines, including IL-1, IL-2, IL-6, IL-17, IL-1β, and the decreased levels of anti-inflammatory cytokines, such as IL-4, IL-10, IL-13, are strongly associated with PSD (8–11). However, the research findings are elusively. Some studies reported that the inflammatory cytokines levels of PSD patients showed no significant changes compared to post-stroke non-depressed (PSND) patients (8). Therefore, identifying the biomarkers that helps to distinguish between PSD and PSND patients is particularly important.

Upon the above concerns, this study aims to find possible biomarkers via multiple bioinformatic approaches. Two GEO datasets, including patients with major depression disease (MDD) and controls (CON), ischemic stroke (IS) patients and CON, were used to analyzed differentially expressed genes (DEGs) and perform enrichment analysis. We then constructed Protein-protein interaction (PPI) network analysis and Random Forest (RF) analysis to screen the candidate hub gene. The immune infiltration between patients and CON was analyzed using Cibersort. We analyzed the proteins that interact with the candidate genes using string database, RNA interactions network of the candidate genes using RNAInter, miRWalk, miRDB and Starbase databases, and the drugs that regulate the candidate genes by DSigDB database. Finally, we further verified the expression of the candidate genes using Quantitative Real-Time PCR from the blood of patients and CON.

## 2 Methods

### 2.1 Data sources

The GSE98793 and GSE16561 datasets were screened from the GEO database using the keywords (depression and peripheral blood, ischemic stroke and peripheral blood),respectively. The GSE98793 dataset contains 192 samples. In our analysis, we used a subset of 128 samples: the 64 CON and the 64 MDD patients without anxiety. The 64 MDD patients with comorbid anxiety were excluded. The GSE16561 dataset contains peripheral blood samples from 39 IS patients and 24 CON.

### 2.2 Differentially expression analysis

The “limma” package in R software was used to screen the DEGs between MDD and CON, IS and CON in the two datasets (p <0.05, |logFC|>0.1), respectively. It is then visualized with heat maps and volcanic maps. Finally, the up-regulated and down-regulated genes in the two datasets were intercrossed by Venn diagram to obtain the differential genes related to both diseases.

### 2.3 Kyoto Encyclopedia of Genes and Genomes pathway enrichment and Gene Ontology analysis

The DEGs were analyzed by KEGG pathway enrichment and GO analysis. GO analysis includes three categories: biological process (BP), cell component (CC) and molecular function (MF).

### 2.4 Protein-protein interaction network analysis

PPI network analysis of DEGs was performed using the STRING online website (https://string-db.org/). Then the results from STRING database were imported into Cytoscape for visualization, and cytoHubba was used to identify the hub genes in the PPI network. The top 10 genes were identified by four algorithms, including “Degree”, “EPC”, “MCC” and “MNC”, and then take the intersection to get the candidate genes.

### 2.5 Random Forest algorithm

The two datasets were analyzed using RF algorithm to screen the top 20 genes, and then the selected genes were interleaved with the genes obtained in PPI to obtain candidate hub genes.

### 2.6 Candidate hub gene expression and Receiver Operating Characteristic analysis

The expression of candidate hub genes in both datasets was analyzed, and then ROC analysis was performed on both datasets to evaluate the sensitivity of the candidate hub gene.

### 2.7 Construction of interacting genes and competing endogenous RNAs networks

The string database was used to find the genes that interacted with the candidate hub genes. RNAInter (http://www.rnainter.org/) database, miRWalk (http://mirwalk.umm.uni-heidelberg.de/) database and miRDB (https://mirdb.org/) database were used to screen the miRNA that interacted with the candidate hub genes, and then Venn diagram was used to find out the co-interacting miRNA. Starbase was used to identify the circRNA interacting with the predicted miRNA, and then Cytoscape was used to visualize the ceRNA network of the interacting circRNA-miRNA-mRNA.

### 2.8 Candidate drug screening

We screened the candidate drugs that target the candidate hub genes using DSigDB (http://tanlab.ucdenver.edu/DSigDB) database.

### 2.9 Immune filtration analysis

Based on gene expression in each sample, the “CIBERSORT” package in R software was used to analyze the proportion of 22 immune cells in each sample. The differences in immune cells among different groups were calculated and visualized. The expression of immune cells and immune checkpoint was analyzed. We also analyzed the correlation between the expression of the candidate hub gene and immune checkpoint.

### 2.10 Participants

All participants (40-70 years old) underwent an initial clinical assessment, including the collection of clinical and demographic information. Depression symptoms in post-stroke patients were evaluated by the Hamilton Depression Rating Scale 17-item (HAMD-17) at one month after stroke by a trained neurologist. A score of 0–7 was considered normal, while a HAMD score > 7 is indicative of depression. Stroke severity was measured using the National Institute of Health Stroke Scale (NIHSS). All MDD patients were hospital-diagnosed according to the International Classification of Disease 10. The inclusion criteria for MDD are specified below: (1) age, 40–70 years; (2) HAMD-17 scores > 7; (3) MDD patients consisted of two scenarios, a first episode not previously treated with any antidepressant medication, or a history of MDD with symptomatic relief on antidepressant medication and cessation of any antidepressant medication for at least one month. The exclusion criteria for all participants are specified below: (1) pregnancy and pregnant females; (2) serious other neurological diseases and tumors, kidney and cardiovascular diseases; (3) participants with any signs of infection. Whole blood samples were collected upon admission. Plasma was then extracted and stored at -80°C.

This study was approved by the Research Ethics Committee of the Affiliated Nanjing Brian Hospital, Nanjing Medical University. Written informed consent was acquired from all participants.

### 2.11 RNA isolation and Quantitative real-time PCR

The plasm RNA from patients and CON was extracted using TRIzol reagent (Invitrogen, Carlsbad, CA, USA). The synthesis of cDNA was achieved using HiScript IV RT SuperMix for qPCR (R423-01, Vazyme). The Taq Pro Universal SYBR qPCR Master Mix (Q712-02, Vazyme) was used to detect the expression of cDNA.

### 2.12 Statistical analysis

R software (v4.3.1) was used for all statistical analysis. The differences among four groups were evaluated using one-way analysis of variance (ANOVA) followed by Tukey *post hoc* tests, T-tests and χ2-tests. The quantitative data are presented as means ± SEM using Graphpad Prism9 or SPSS20.0 software (SPSS, Inc., Chicago, IL, USA). The p-value < 0.05 was considered statistically significant.

## 3 Results

### 3.1 Identification of DEGs in two datasets

**Figure 1:**
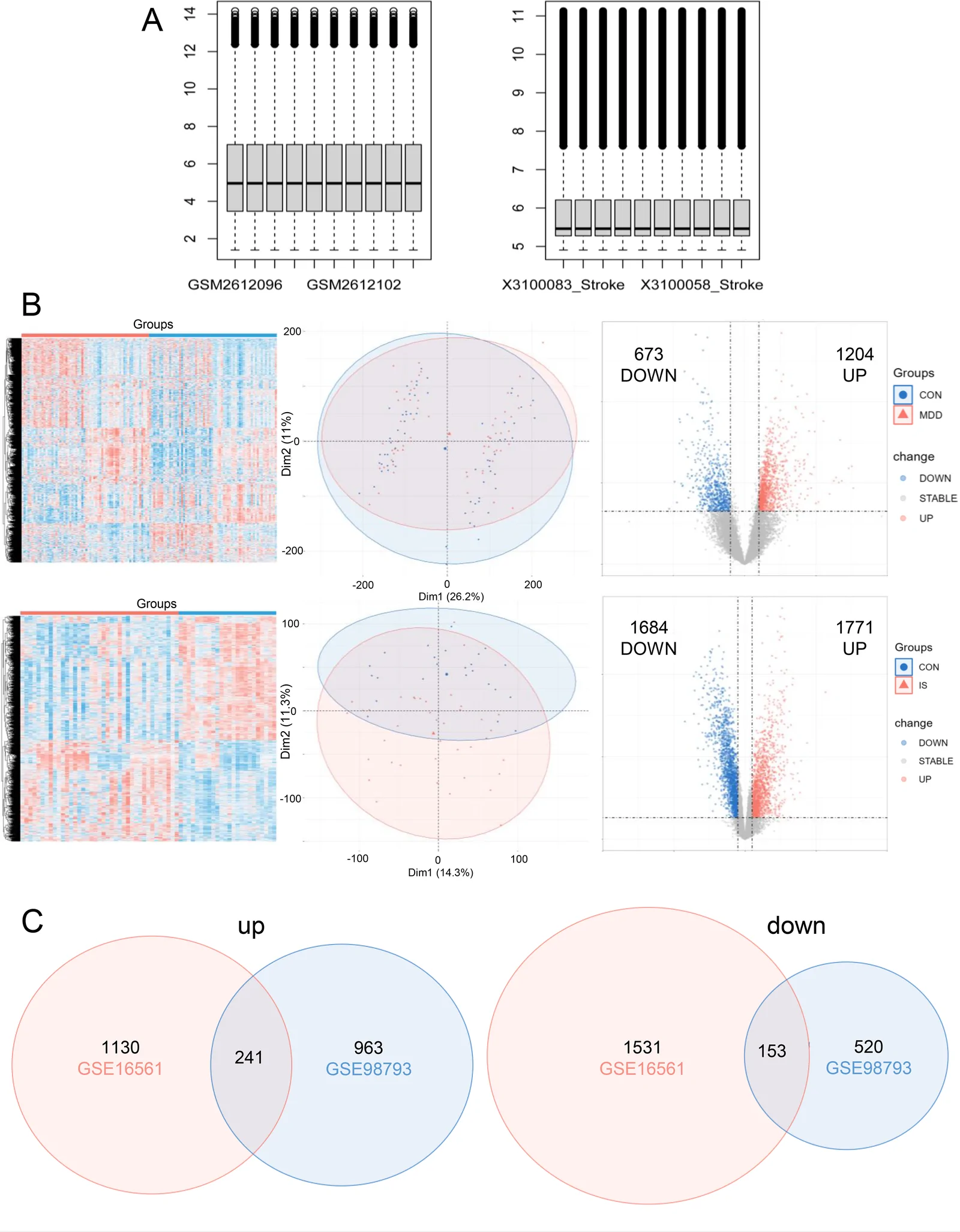
Differentially expressed genes (DEGs) screening. **(A)** The expression of mRNA in GSE98793 and GSE16561; **(B)** Heatmap, venn, and volcano plot of in GSE98793 and GSE16561, the thresholds applied in the volcano plot analysis: p < 0.05 and |log_2_ fold change| > 0.1; **(C)** Venn diagram illustrated the number of shared and unique DEGs.

The mRNA expression of the two data sets is shown in **Figure 1A**. In GSE98793, 1204 genes were upregulated and 673 genes were downregulated in MDD compared to the CON (**Figure 1B**). 1684 down-regulated genes and 1371 up-regulated genes were screened in GSE16561 (**Figure 1B**). Then the significantly up-regulated and down-regulated genes of the two datasets were intersected, respectively, and a total of 241 up-regulated genes and 153 down-regulated genes were screened (**Figure 1C**).

### 3.2 Gene Ontology enrichment analysis

**Figure 2:**
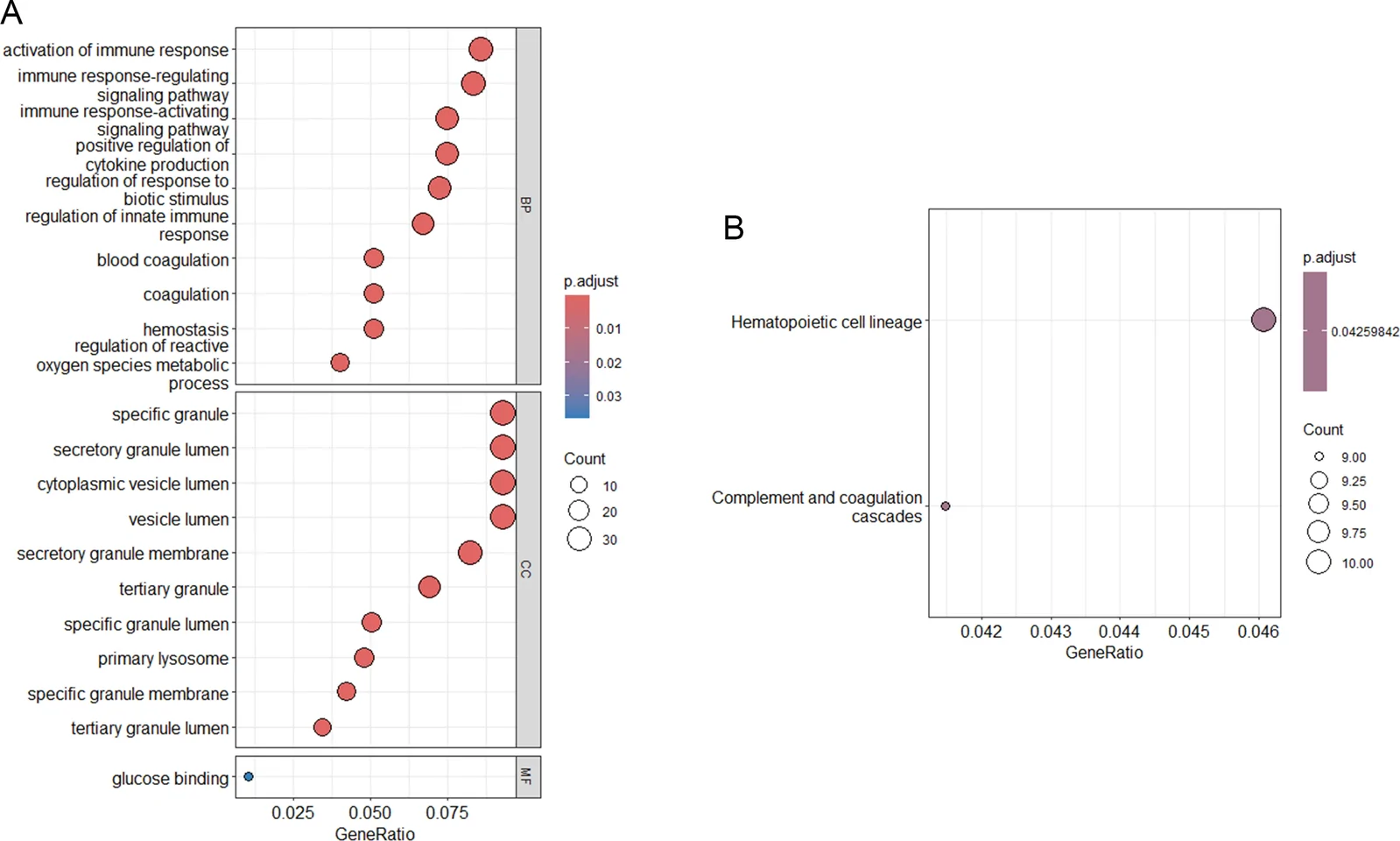
Functional enrichment analysis. **(A)** KEGG pathway analysis of DEGs; **(B)** GO functional analysis of DEGs.

GO analysis of the DEGs was showed in **Figure 2A**. The BP category was enriched in activation of immune response, immune response-regulating signaling pathway, immune response-activating signaling pathway, positive regulation of cytokine production, regulation of response to biotic stimulus, regulation of innate immune response, blood coagulation, coagulation, hemostasis, and regulation of reactive oxygen species metabolic process. For the CC, enrichment was seen in specific granule, secretory granule lumen, cytoplasmic vesicle lumen, vesicle lumen, secretory granule membrane, tertiary granule, specific granule lumen, primary lysosome, specific granule membrane and tertiary granule lumen. The MF for these DEGs was glucose integrin binding. KEGG analyses revealed that the DEGs were involved in hematopoietic cell lineage, and complement and coagulation cascades (**Figure 2B**).

### 3.3 Exploration of core genes

**Figure 3:**
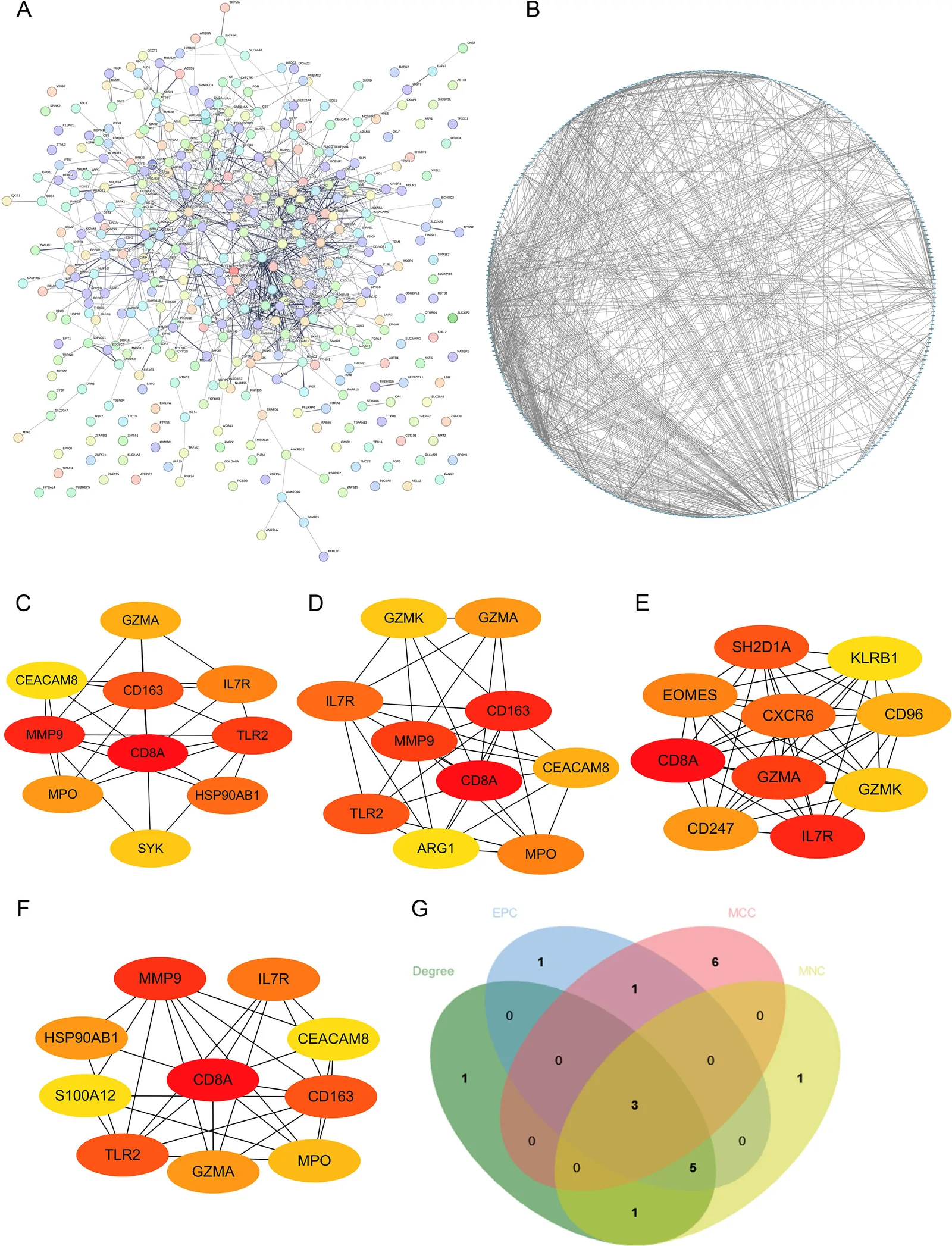
Construction of PPI network. **(A, B)** The visualization of PPI network analysis of DEGs; the analysis of top 10 DEGs with Degree **(C)**, EPC **(D)**, MCC **(E)** and MNC **(F)** algorithms. **(G)** Venn diagram showed the number of shared and unique DEGs of the four algorithms.

To explore core genes from the DEGs, the DEGs were uploaded to STING database for PPI network analysis (**Figure 3A**), and then the STRING results were imported into Cytoscape for visualization (**Figure 3B**).

Four algorithms (Degree, EPC, MCC and MNC) in Cytoscape were utilized to calculate the hub genes (**Figures 3C–F**). CD8A, IL7R and GZMA were identified as the potential hub genes (**Figure 3G**).

### 3.4 RF algorithm

**Figure 4:**
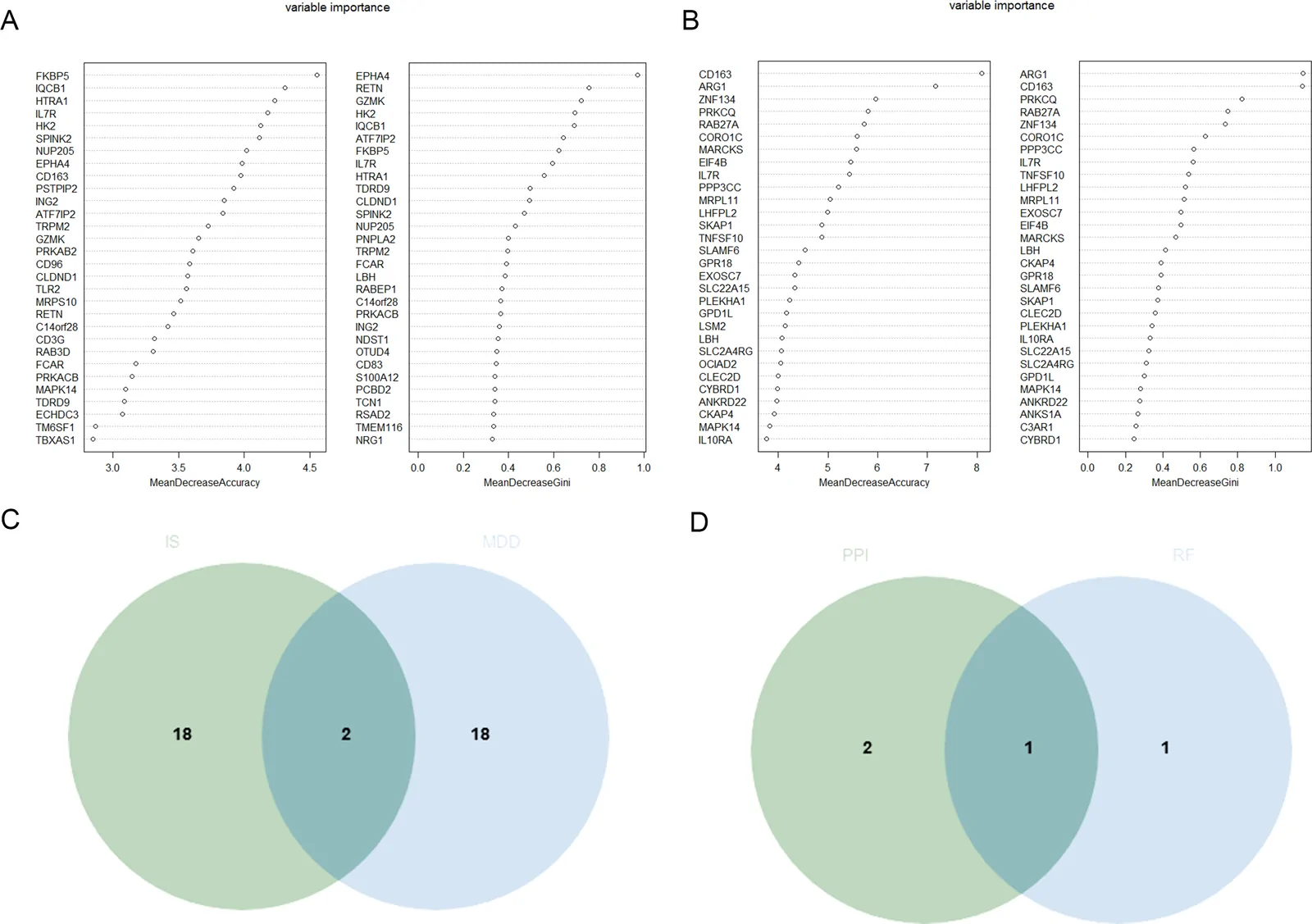
Identification of the candidate hub DEGs. **(A)** The candidate hub DEGs was performed through RF in GSE98793 **(A)** and GSE16561 **(B)**; **(C)** Venn diagram illustrated the number of shared and unique DEGs screened from GSE98793 and GSE16561; **(D)** Venn diagram showed the candidate hub DEGs screened between RF and above four algorithms.

RF algorithm was used to screen the intersection of the top 20 potential genes of the two datasets, respectively (**Figures 4A, B**). And then, the intersection genes obtained by RF algorithm and the three candidate genes mentioned above were intersected, leading to the identification of IL-7R as a potential diagnostic marker (**Figures 4C, D**).

### 3.5 Expression level and diagnostic value of characteristic biomarkers in MDD

**Figure 5:**
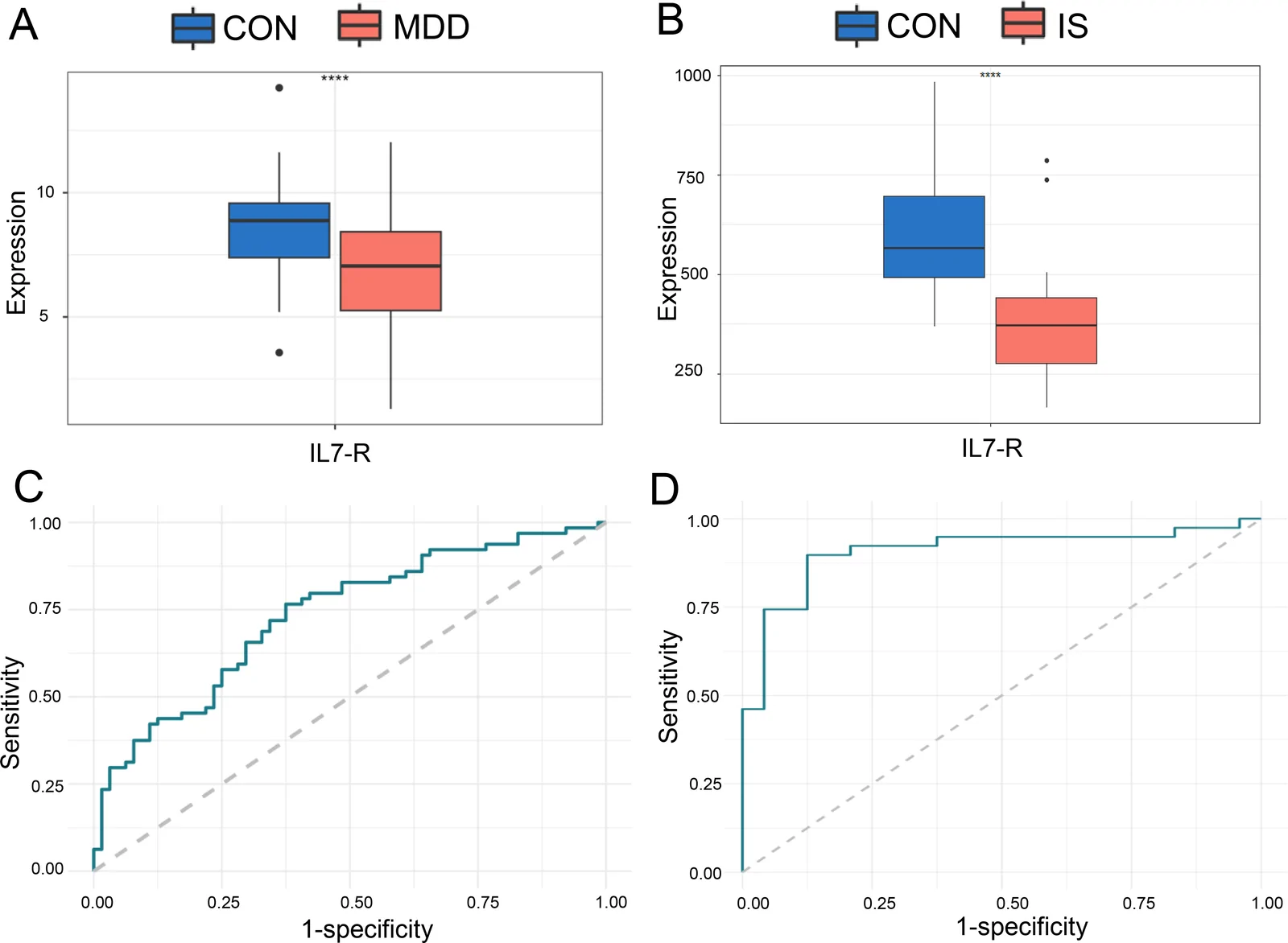
The ROC curve of the candidate hub DEGs in MDD and IS patients. The expression of IL-7R in GSE98793 **(A)** and GSE16561 **(B)**; ROC analysis of IL-7R between IS **(C)** or MDD **(D)** patients and CON. *** p < 0.001.

IL-7R expression levels were significantly lower in MDD patients than in CON in GSE98793 dataset (**Figure 5A**). And IL-7R expression levels also were significantly lower in IS patients than in CON in GSE16561 dataset (**Figure 5B**).

Based on these two data sets, ROC analysis was then performed on IL-7R. The results showed that IL-7R had a good diagnostic and predictive effect on MDD patients and IS patients, with AUC of 0.7332 of GSE98793 and 0.9081 of GSE16561, respectively (**Figures 5C, D**).

### 3.6 The related gene-gene interaction network

**Figure 6:**
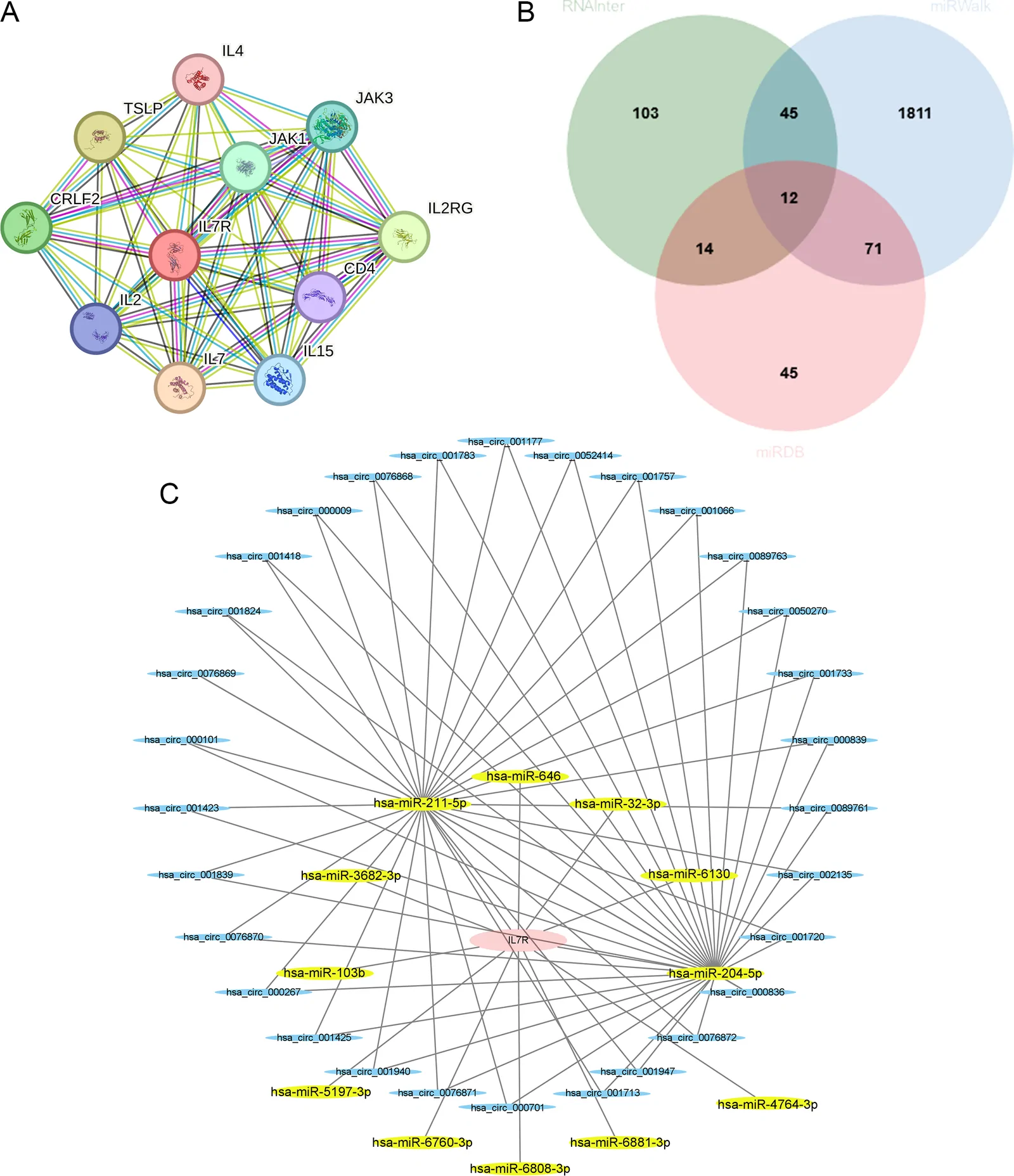
Relative prediction about IL-7R. **(A)** Construction of interacting genes with IL-7R; **(B)** The predicted miRNA that interacted with IL-7R in three databases; **(C)** The ceRNA networks. IL-7R are colored in red, miRNAs are colored in yellow, and circRNAs are colored in blue.

We constructed the IL7R-related gene-gene interaction network using String database, and the results revealed that 10 central genes are connected to IL-7R (**Figure 6A**).

Using the RNAInter, miRWalk and miRDB, we found 9 miRNA that can interact with IL-7R, including hsa-miR-204-5p, hsa-miR-211-5p, hsa-miR-32-3p, hsa-miR-3682-3p, hsa-miR-5197-3p, hsa-miR-6760-3p, hsa-miR-6808-3p, hsa-miR-6881-3p and hsa-miR-4764-3p (**Figure 6B**). Then we used ENCORI database to explore the circRNA that can regulate miRNA and the circRNA-miRNA-mRNA regulatory network was constructed by Cytoscape software (**Figure 6C**).

### 3.7 Potential target drugs

We found the perturbagen signatures and computational drug signatures that can target IL-7R, which shown in **Supplementary Tables S1**, **S2** (In the **Supplementary Materials**) by DSigDB database. In total, we found 7 and 22 drugs that could increase and decrease IL-7R expression in **Supplementary Table S1**. In addition, there are 32 drug signatures extracted from literatures using a mixture of manual curation and by automatic computational approaches in **Supplementary Table S2**.

### 3.8 Immune infiltration analysis

**Figure 7:**
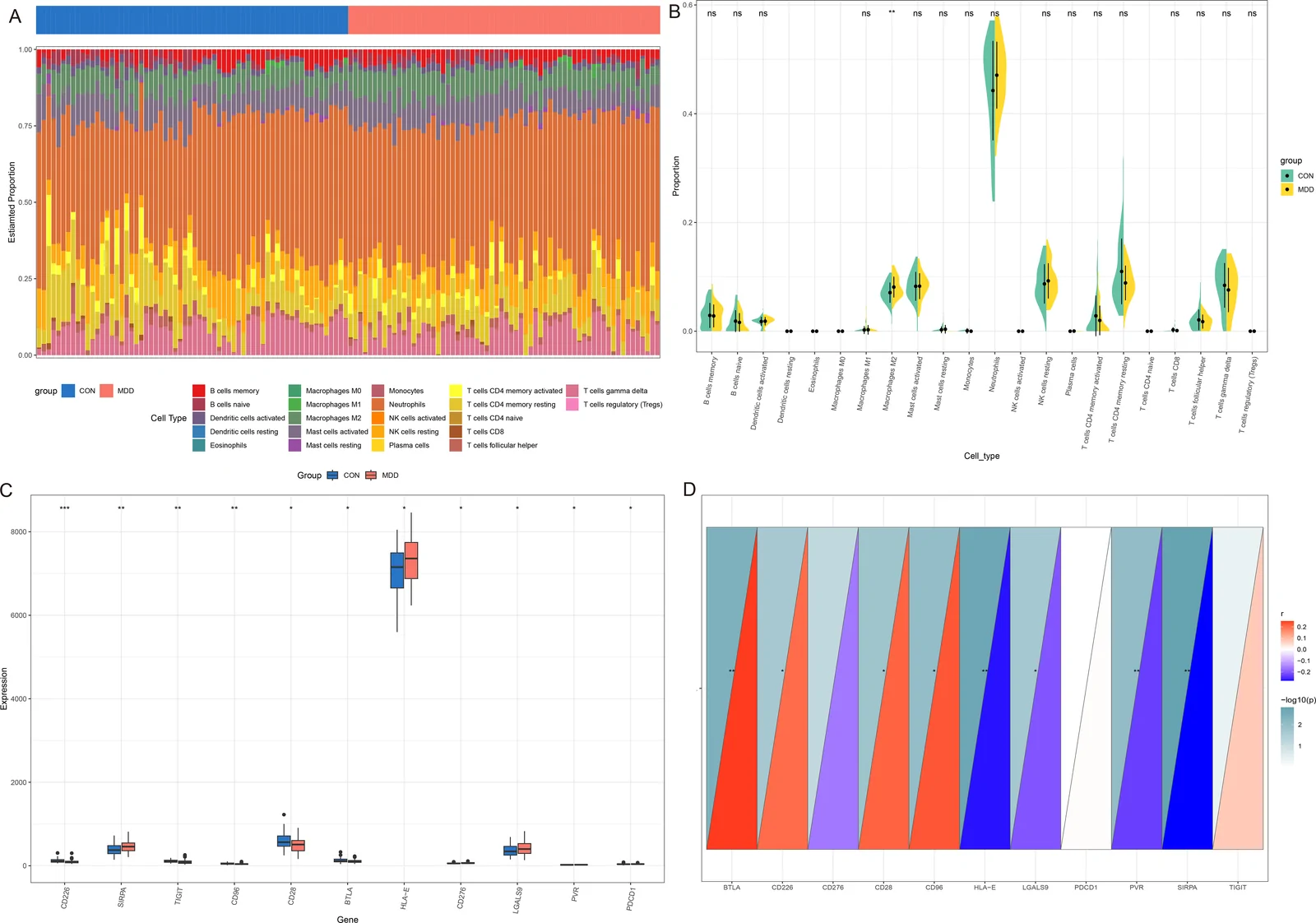
Immune infiltration analysis between MDD and CON. **(A)** The heatmap of relative abundance of 22 immune cells; **(B)** Cello diagram of immune infiltration of 22 immune cells; **(C)** Boxplot of immune checkpoint; **(D)** Pearson correlation analysis between immune cells and IL-7R. ns: no significance, * p < 0.05, ** p < 0.01, ***p < 0.001.

The CIBERSORT was used to analyze the proportion of 22 types of immune cells in peripheral blood from two datasets. In GSE98793, the proportions of macrophages M0 and monocytes in MDD patients were significantly higher than those in CON. However, the proportion of eosinophils, T cells CD4 memory resting and T cells gamma delta in MDD was significantly lower than that in CON (**Figures 7A, B**). Next, we analyzed the differences in immune checkpoint gene expression between MDD patients and CON. Compared with CON, there are significant differences 11 gene expression, including 4 high expressed genes (SIRPA, HLA - E and LGALS9, etc.) and 7 lower expressions (CD226, TIGIT and CD96, etc., **Figure 7C**). Finally, the correlation between IL-7R and the above immune checkpoint genes was analyzed. The results showed that IL-7R was positively correlated with BTLA, CD226, CD28 and CD96, and negatively correlated with HLA-E, LGALS9, PVR and SIRPA (**Figure 7D**).

**Figure 8:**
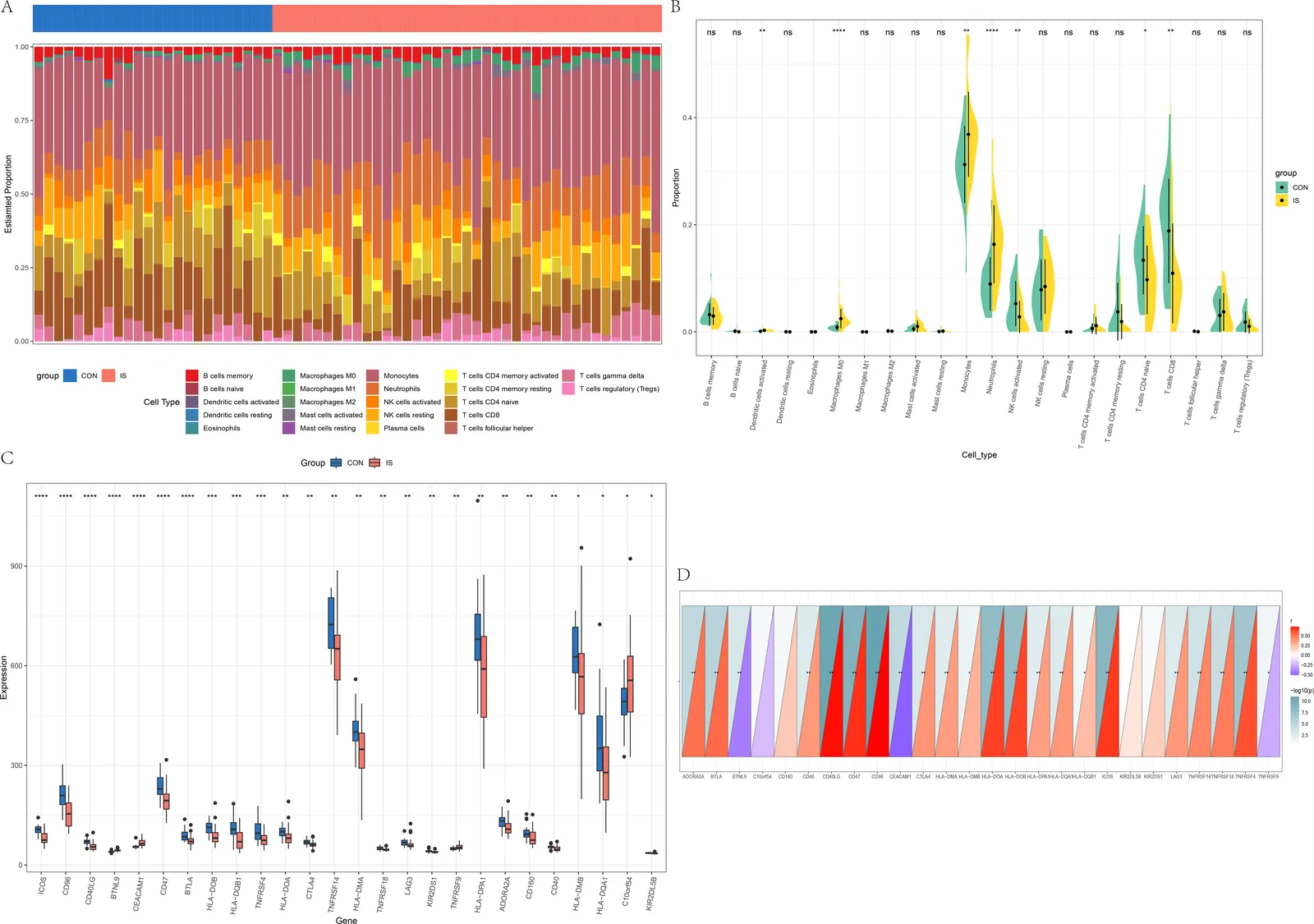
Immune infiltration analysis between IS and CON. **(A)** The heatmap of relative abundance of 22 immune cells; **(B)** Cello diagram of immune infiltration of 22 immune cells; **(C)** Boxplot of immune checkpoint; **(D)** Pearson correlation analysis between immune cells and IL-7R. ns: no significance, * p < 0.05, ** p < 0.01, ***p < 0.001.

In GSE16561, we found that Dendritic cells activated, Macrophages M0, Monocytes, and Neutrophils were the most significantly increased, and NK cells resting, T cells CD4 naïve and T cells CD6 were the most significantly decreased in IS patients, compared with CON (**Figures 8A, B**). The differences in immune checkpoint gene expression between MDD patients and CON were shown in **Figure 8C**. The correlation between IL-7R and the above immune checkpoint genes were presented in **Figure 8D**. There are 19 immune checkpoint genes are positively correlated with IL-7R and 3 immune checkpoint genes are negatively with IL-7R.

### 3.9 Demographic and clinical characteristics of participants

**Figure 9:**
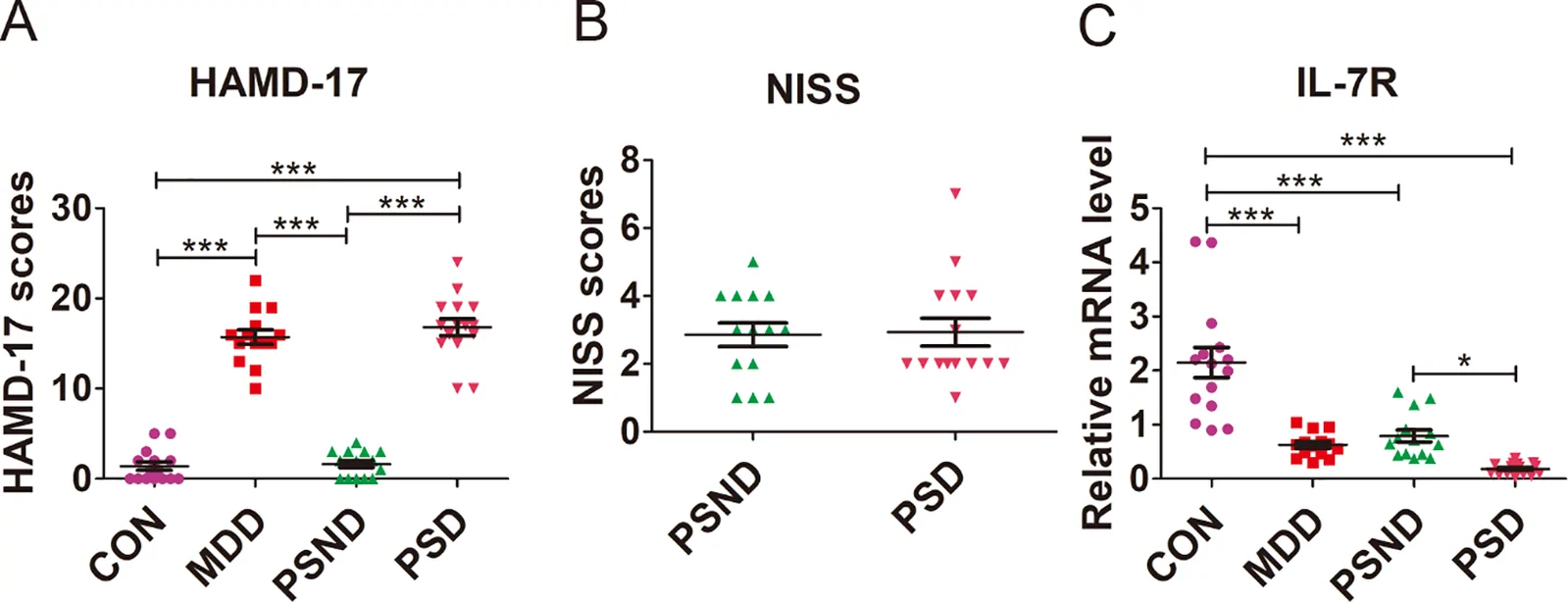
Comparison of **(A)** HAMD-17 scores, **(B)** NISS scores and **(C)** IL-7R expression among groups. CON (n=15): Controls, MDD (n=14): Major depressive disease, PSND (n=14): post-stroke non-depressed, PSD (n=15): Post-stroke depression. ***p<0.001, * p<0.05.

The demographic and clinical characteristics of patients and CON are summarized in **Supplementary Table S3**. There were no significant differences in age (F = 1.416, p _ANOVA_ = 0.248), sex (χ2 = 1.7702, p = 0.636), education level (F = 2.554, p _ANOVA_ = 0.065) and percentage of smokers (χ2 = 3.411, p = 0.332), drinkers (χ2 = 3.095, p = 0.377), hypertension patients (χ2 = 3.416, p = 0.3332) among the MDD (n = 14), PSND (n = 14), PSD (n =15) and CON (n =15) groups. The percentage of diabetic patients in the PSND and PSD groups was higher than in the CON and MDD groups (χ2 = 10.255, p = 0.017), but there was no significant statistical difference between the PSND and PSD groups. No significant difference was found in the NIHSS scores (t = -0.142, p = 0.888) between the PSND and PSD groups (**Figure 9B**). The HAMD-17 scores (F = 152.408, p _ANOVA_ < 0.001) of the MDD (MDD vs CON: p < 0.001, MDD vs PSND: p < 0.001) and PSD (PSD vs CON: p < 0.001, PSD vs PSND: p < 0.001) groups were significantly higher than those of the CON and PSND groups, but there was no significant statistical difference between the MDD and PSD groups (**Figure 9A**, p = 0.685).

### 3.10 The validation of the potential diagnostic marker

We examined the IL-7R expression in the plasm of MDD, PSD, PSND and CON groups (**Figure 9C**, F = 30.053, p _ANOVA_ < 0.001). We found that the expression of IL7R was significantly lower in MDD (p _Turkey_ < 0.001), PSD (p _Turkey_ < 0.001) and PSND (p _Turkey_ < 0.001) groups than in the CON group. The expression of IL-7R in PSD group was lower than that in PSND group (p _Turkey_ = 0.041).

## 4 Discussion

In the present study, by screening and enrichment analysis of gene expression profiles of depression and stroke-related datasets, 394 key genes involved in both disorders were obtained. The expression of IL-7R in patients with both diseases and comorbid conditions was further confirmed by bioinformatics and blood sample validation. The findings may provide new directions for elucidating the molecular mechanisms of post-stroke inflammation.

IL-7R is composed of a specific α chain (IL-7Rα, CD127) and a common γ chain (IL-7Rγ, CD132) (12), it is broadly expressed in the lymphoid system: on B cell progenitors, innate lymphoid cells and throughout the development and maturation of T cell (12, 13). IL-7R receives IL-7 mediated signals that promote anti-apoptosis and stimulate proliferation, playing an important role in maintaining normal immune function (14). Once the IL-7/IL-7R signaling pathway is activated, it can induce the differentiation of B cell progenitors into mature B cells, promote the proliferation and differentiation of thymocytes, and induce the proliferation and differentiation of pro-T cells into effector T cells, thereby playing a pro-inflammatory role (12, 15). Due to the fact that mature B cells, selecting thymocytes, and effector T cells do not express IL-7R, the activation of the IL-7/IL-7R pathway may lead to a decrease in IL-7R expression (15, 16). This characteristic makes IL-7R a measurable indicator of immune activity. A view holds that as a homeostatic cytokine regulating T cells function, IL-7/IL-7R signaling pathway is involved in the cascade possibly contributing to the hypothesized depression-related lymphokine suppression (17, 18). However, previous studies found that the levels of IL-7 are elusively in depression patients. One research reported that levels of IL-7 is elevated in depressed subjects (19), while some studies found that the depression patients have significantly lower levels of IL-7 than CON (20, 21). It hypothesized that the different findings of IL-7 may be due to its dynamic changes in the process of regulating immune homeostasis (17, 22). While the expression of IL-7R significantly decreases after IL-7/IL-7R signaling pathway activation, which may make it a more stable indicator. In this study, we found that IL-7R expression levels were significantly lower in MDD patients than in CON in GSE98793 dataset. The data from patient validation also showed that IL-7R expression levels were significantly lower in MDD group than in CON group, suggesting that the decreased IL-7R level may be a characteristic change in MDD.

Neuroinflammation is crucially involved in the pathophysiology of stroke (23). Once stroke occurs, lesioned area of brain initiates cell death, leading to the uncontrolled release of intracellular molecules (24). These molecules activate sterile inflammation, which leads to the occurrence of secondary brain injury (25). Although the exact mechanism of PSD is still unclear, the inflammatory theory indicates that PSD may be related to an overactivated inflammatory response (26). The “cytokine hypothesis” suggests that the overactivated inflammatory response can produce a mass of pro-inflammatory cytokines, such as IL-1, IL-2, IL-6, IL-17, IL-1β (8, 27). These cytokines interact with 5-HT, resulting in the amplification of inflammatory processes and activation of indoleamine-2,3-dioxygenase (IDO) in the limbic lobe (28). Then IDO converts tryptophan into kynurenine, causing a depletion of 5-HT in the paralimbic structures. This physiological dysfunction may contribute to the development of PSD (1, 29). To the best of our knowledge, the changes of IL-7/IL-7R signaling pathway in PSD remain to be elucidated. Previous study suggested that the levels of IL-7 were decreased in severe stroke patients, and the decrease in IL-7 levels is associated with poor prognosis in patients (30, 31). Consistent with these findings, we found that the expression levels of IL-7R were significantly lower in stroke patients compared to the CON. We further demonstrated that the expression of IL-7R in mRNA levels of PSD patients is lower than that of PSND patients. We speculate that the lower levels of IL-7R in PSD patients may be associated with the overactivated inflammatory response according to the inflammatory theory. When the immune system is overactivated in the PSD patients, B cell progenitors are induced into mature B cells, pro-T cells are profiled into effector T cells, which leads to the downregulation of IL-7R expression.

This study has several limitations. First, a large sample is needed to validate our findings. Second, follow up studies are needed to confirm whether IL-7R has prognostic value for the early diagnosis of PSD. Third, although this study identified some potential target drugs that can upregulate IL-7R expression, further experiments are needed to verify whether these drugs can improve symptoms associated with PSD.

## 5 Conclusion

In conclusion, the present study puts out the hypothesis that due to the overactivated inflammatory response, the IL-7R levels of PSD patients are lower than that of PSND patients. IL-7R may serve as an early diagnostic marker to distinguish between PSD and PSND patients, and targeting IL-7R as a therapeutic target could potentially improve treatment outcomes for PSD in the future.

## Data Availability

Publicly available datasets were analyzed in this study. This data can be found here: GSE98793 and GSE16561. The original contributions presented in the study are included in the article/**Supplementary Material**. Further inquiries can be directed to the corresponding author.
