# Correction: New insights into roles of IL-7R gene as a diagnostic biomarker for post-stroke depression

**DOI:** 10.3389/fimmu.2026.2000379

**Published:** 2026-10-09

**Authors:** Mengyu Liu, Haochen Sun, Qun Yao, Duohao Wang, Jihong Zhang, Xing Ye, Xinyang Qi

**Affiliations:** 1 Department of Neurology, Affiliated Nanjing Brain Hospital, Nanjing Medical University, Nanjing, China; 2 Department of Neurology, School of Medicine, Southeast University, Nanjing, China

**Keywords:** stroke, post-stroke depression, major depression, neuroinflammation, IL-7R

There was a mistake in the caption of figure **Figure 1B** as published. The thresholds applied in the volcano plot analysis were not specified. The corrected caption of figure **Figure 1B** appears below.

“**(B)** Heatmap, venn, and volcano plot of in GSE98793 and GSE16561, the thresholds applied in the volcano plot analysis: p < 0.05 and |log_2_ fold change| > 0.1;”

Also, In the **2 Methods**, *2.1 Data sources* section, the sample composition and exclusion criteria of the GSE98793 dataset are not explicitly stated.

A correction has been made to the section **2 Methods**, *2.1 Data sources*, paragraph 1:

“The GSE98793 and GSE16561 datasets were screened from the GEO database using the keywords (depression and peripheral blood, ischemic stroke and peripheral blood), respectively. The GSE98793 dataset contains 192 samples. In our analysis, we used a subset of 128 samples: the 64 CON and the 64 MDD patients without anxiety. The 64 MDD patients with comorbid anxiety were excluded. The GSE16561 dataset contains peripheral blood samples from 39 IS patients and 24 CON.”

The original version of this article has been updated.

