# Supplementary material for: New insights into roles of IL-7R gene as a diagnostic biomarker for post-stroke depression
Source: Front Immunol. 2024 Dec 23;15:1506214. doi: 10.3389/fimmu.2024.1506214 (PMC11700794; doi:10.3389/fimmu.2024.1506214)
Supplement: Supplementary file 2 [file Table1.docx]

Table S1 The predicted perturbagen signatures that can target IL-7R

| **Gene** | **Source** | **Chemical Name** |
| --- | --- | --- |
| IL-7R | DOWN | 5707885 |
|  |  | [etifenin](https://dsigdb.tanlab.org/DSigDBv1.0/displayDrug.py?db=d3&id=740) |
|  |  | [LY-294002](https://dsigdb.tanlab.org/DSigDBv1.0/displayDrug.py?db=d3&id=1088) |
|  |  | neostigmine bromide |
|  |  | [scriptaid](https://dsigdb.tanlab.org/DSigDBv1.0/displayDrug.py?db=d3&id=1692) |
|  |  | [trichostatin A](https://dsigdb.tanlab.org/DSigDBv1.0/displayDrug.py?db=d3&id=1900) |
|  |  | [vorinostat](https://dsigdb.tanlab.org/DSigDBv1.0/displayDrug.py?db=d3&id=1972) |
|  | UP | [(-)-isoprenaline](https://dsigdb.tanlab.org/DSigDBv1.0/displayDrug.py?db=d3&id=7) |
|  |  | [alprostadil](https://dsigdb.tanlab.org/DSigDBv1.0/displayDrug.py?db=d3&id=151) |
|  |  | [alvespimycin](https://dsigdb.tanlab.org/DSigDBv1.0/displayDrug.py?db=d3&id=160) |
|  |  | [anisomycin](https://dsigdb.tanlab.org/DSigDBv1.0/displayDrug.py?db=d3&id=193) |
|  |  | [cicloheximide](https://dsigdb.tanlab.org/DSigDBv1.0/displayDrug.py?db=d3&id=435) |
|  |  | [geldanamycin](https://dsigdb.tanlab.org/DSigDBv1.0/displayDrug.py?db=d3&id=851) |
|  |  | [lomustine](https://dsigdb.tanlab.org/DSigDBv1.0/displayDrug.py?db=d3&id=1073) |
|  |  | [loxapine](https://dsigdb.tanlab.org/DSigDBv1.0/displayDrug.py?db=d3&id=1081) |
|  |  | [menadione](https://dsigdb.tanlab.org/DSigDBv1.0/displayDrug.py?db=d3&id=1123) |
|  |  | [MG-262](https://dsigdb.tanlab.org/DSigDBv1.0/displayDrug.py?db=d3&id=1198) |
|  |  | [monorden](https://dsigdb.tanlab.org/DSigDBv1.0/displayDrug.py?db=d3&id=1227) |
|  |  | [parthenolide](https://dsigdb.tanlab.org/DSigDBv1.0/displayDrug.py?db=d3&id=1383) |
|  |  | [phenoxybenzamine](https://dsigdb.tanlab.org/DSigDBv1.0/displayDrug.py?db=d3&id=1433) |
|  |  | [pizotifen](https://dsigdb.tanlab.org/DSigDBv1.0/displayDrug.py?db=d3&id=1480) |
|  |  | [profenamine](https://dsigdb.tanlab.org/DSigDBv1.0/displayDrug.py?db=d3&id=1561) |
|  |  | [promethazine](https://dsigdb.tanlab.org/DSigDBv1.0/displayDrug.py?db=d3&id=1570) |
|  |  | [puromycin](https://dsigdb.tanlab.org/DSigDBv1.0/displayDrug.py?db=d3&id=1589) |
|  |  | [sanguinarine](https://dsigdb.tanlab.org/DSigDBv1.0/displayDrug.py?db=d3&id=1676) |
|  |  | [tanespimycin](https://dsigdb.tanlab.org/DSigDBv1.0/displayDrug.py?db=d3&id=1802) |
|  |  | [triprolidine](https://dsigdb.tanlab.org/DSigDBv1.0/displayDrug.py?db=d3&id=1921) |
|  |  | [vinburnine](https://dsigdb.tanlab.org/DSigDBv1.0/displayDrug.py?db=d3&id=1966) |
|  |  | [withaferin A](https://dsigdb.tanlab.org/DSigDBv1.0/displayDrug.py?db=d3&id=1973) |

| **Gene** | **Source** | **Chemical Name** |
| --- | --- | --- |
| IL-7R | Curation of targets from Comparative Toxicogenomics Database (CTD). | [(-)-Epigallocatechin gallate](https://dsigdb.tanlab.org/DSigDBv1.0/displayDrug.py?db=d4_ctd&id=2033) |
|  |  | [1-chloro-2,4-dinitrobenzene](https://dsigdb.tanlab.org/DSigDBv1.0/displayDrug.py?db=d4_ctd&id=5848) |
|  |  | [2-Mercaptobenzothiazole](https://dsigdb.tanlab.org/DSigDBv1.0/displayDrug.py?db=d4_ctd&id=240) |
|  |  | [2-Nonenal, 4-hydroxy-, (2E,4R)-](https://dsigdb.tanlab.org/DSigDBv1.0/displayDrug.py?db=d4_ctd&id=1295) |
|  |  | [4-Hydroxytamoxifen](https://dsigdb.tanlab.org/DSigDBv1.0/displayDrug.py?db=d4_ctd&id=850) |
|  |  | [8-Bromo-cAMP, Na](https://dsigdb.tanlab.org/DSigDBv1.0/displayDrug.py?db=d4_ctd&id=7044) |
|  |  | [ARSENIC](https://dsigdb.tanlab.org/DSigDBv1.0/displayDrug.py?db=d4_ctd&id=5442) |
|  |  | [aspirin](https://dsigdb.tanlab.org/DSigDBv1.0/displayDrug.py?db=d4_ctd&id=5447) |
|  |  | [Bortezomib](https://dsigdb.tanlab.org/DSigDBv1.0/displayDrug.py?db=d4_ctd&id=3736) |
|  |  | [Cadmium sulfate](https://dsigdb.tanlab.org/DSigDBv1.0/displayDrug.py?db=d4_ctd&id=1745) |
|  |  | [calcitriol](https://dsigdb.tanlab.org/DSigDBv1.0/displayDrug.py?db=d4_ctd&id=5558) |
|  |  | [cinnamaldehyde](https://dsigdb.tanlab.org/DSigDBv1.0/displayDrug.py?db=d4_ctd&id=671) |
|  |  | [Cobalt sulfate](https://dsigdb.tanlab.org/DSigDBv1.0/displayDrug.py?db=d4_ctd&id=1238) |
|  |  | [COPPER](https://dsigdb.tanlab.org/DSigDBv1.0/displayDrug.py?db=d4_ctd&id=5706) |
|  |  | [cyclosporin A](https://dsigdb.tanlab.org/DSigDBv1.0/displayDrug.py?db=d4_ctd&id=7121) |
|  |  | [Decitabine](https://dsigdb.tanlab.org/DSigDBv1.0/displayDrug.py?db=d4_ctd&id=750) |
|  |  | [Demecolcine](https://dsigdb.tanlab.org/DSigDBv1.0/displayDrug.py?db=d4_ctd&id=5762) |
|  |  | [dexamethasone](https://dsigdb.tanlab.org/DSigDBv1.0/displayDrug.py?db=d4_ctd&id=5779) |
|  |  | [estradiol](https://dsigdb.tanlab.org/DSigDBv1.0/displayDrug.py?db=d4_ctd&id=5920) |
|  |  | [eugenol](https://dsigdb.tanlab.org/DSigDBv1.0/displayDrug.py?db=d4_ctd&id=5949) |
|  |  | [folic acid](https://dsigdb.tanlab.org/DSigDBv1.0/displayDrug.py?db=d4_ctd&id=5997) |
|  |  | [formaldehyde](https://dsigdb.tanlab.org/DSigDBv1.0/displayDrug.py?db=d4_ctd&id=6001) |
|  |  | [Mustard gas](https://dsigdb.tanlab.org/DSigDBv1.0/displayDrug.py?db=d4_ctd&id=6356) |
|  |  | [nerol](https://dsigdb.tanlab.org/DSigDBv1.0/displayDrug.py?db=d4_ctd&id=373) |
|  |  | [NICKEL SULFATE](https://dsigdb.tanlab.org/DSigDBv1.0/displayDrug.py?db=d4_ctd&id=1417) |
|  |  | [Pemetrexed](https://dsigdb.tanlab.org/DSigDBv1.0/displayDrug.py?db=d4_ctd&id=3054) |
|  |  | [POTASSIUM CHROMATE](https://dsigdb.tanlab.org/DSigDBv1.0/displayDrug.py?db=d4_ctd&id=1284) |
|  |  | [progesterone](https://dsigdb.tanlab.org/DSigDBv1.0/displayDrug.py?db=d4_ctd&id=6624) |
|  |  | [raloxifene](https://dsigdb.tanlab.org/DSigDBv1.0/displayDrug.py?db=d4_ctd&id=7367) |
|  |  | [resveratrol](https://dsigdb.tanlab.org/DSigDBv1.0/displayDrug.py?db=d4_ctd&id=2483) |
|  |  | [Silica](https://dsigdb.tanlab.org/DSigDBv1.0/displayDrug.py?db=d4_ctd&id=6678) |
|  |  | [Sodium dodecyl sulfate](https://dsigdb.tanlab.org/DSigDBv1.0/displayDrug.py?db=d4_ctd&id=6753) |

Table S2 The computational drug signatures that can target IL-7R

Table S3. Demograpic and clinical characteristics of all subjects

|  | CON (n=15) | MDD (n=14) | PSND (n=14) | PSD (n=15) | P |
| --- | --- | --- | --- | --- | --- |
| Age (yr) | 60.33±1.18 | 60.50±1.23 | 57.93±1.05 | 58.00±1.27 | 0.248 |
| Gender (male/female) | 7/8 | 8/6 | 6/8 | 5/10 | 0.636 |
| Education (yr) | 13.47±0.68 | 11.14±1.63 | 9.71±0.87 | 10.47±0.68 | 0.065 |
| NHISS |  |  | 2.86±0.35 | 2.93±0.41 | 0.888 |
| Hypertension (%) | 13.3% | 14.3% | 35.7% | 33.3% | 0.332 |
| Diabetes (%) | 0.0% | 14.3% | 42.9% | 40.0% | 0.017 |
| Smoke (%) | 13.3% | 21.4% | 35.7% | 40.0% | 0.332 |
| Alcohol (%) | 6.7% | 14.3% | 28.6% | 26.7% | 0.377 |

Data are expressed as mean ± s.e.m.
